# Crystallites and Electric Fields in Solid Ammonia

**DOI:** 10.1002/open.202000118

**Published:** 2020-07-16

**Authors:** Andrew Cassidy, Rachel L. James, Anita Dawes, David Field

**Affiliations:** ^1^ Department of Physics and Astronomy Aarhus University Ny Munkegade 120 8000 Aarhus Denmark; ^2^ School of Physical Sciences The Open University Walton Hall Milton Keynes MK7 6AA UK

**Keywords:** Wannier-Mott exciton, spectroscopy, ammonia, molecular ices, polarised solids

## Abstract

Absorption spectra of vacuum‐deposited films of ammonia have been obtained in the range 115 nm to 310 nm for a set of 15 deposition temperatures, T_d_, between 20 K and 80 K. Results focus upon the region 115 nm to 130 nm in overlapping D, E, F and G←X Rydberg transitions involving Wannier‐Mott excitons. We identify two phases of ammonia, showing the solid to be polymorphic. Peak absorption wavelengths in the region of interest are found to shift to the red by 299 cm^−1^, for T_d_ between 20 K to 50 K, and 1380 cm^−1^ for T_d_ between 55 K to 80 K. Shifts provide evidence for the presence of spontaneously generated electric fields in these films, of values in excess of 10^8^ V m^−1^ for T_d_ of 20 K to 50 K to a few times 10^7^ V m^−1^ for 55 K to 80 K. Results enable us to place a lower limit of 1.58 nm on the size of crystallites in the low temperature regime. This dimension represents 16 unit cells or 64 species, giving a more quantitative description than the nebulous term amorphous, as applied to solid ammonia. We also determine that crystallites formed in the high temperature regime contain, within ±20 %, 1688, 756 and 236 molecules of ammonia, respectively at T_d_ of 65 K, 60 K and 55 K.

## Graphical abstract

**What do we mean** when we describe a film of vacuum‐deposited material as amorphous and what by crystalline? In this study of spontaneously electrical solid ammonia, we show that the so‐called “amorphous” phase, prepared between 20–50 K, is a crystalline material composed of crystallites containing >64 molecules. More broadly, we introduce a technique to quantify the size of crystallites in nanoscale films.

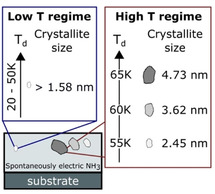

## 1 Introduction

What is conveyed when a material is dubbed amorphous, as opposed to crystalline? We propose here that so‐called amorphous material is composed of nanometre‐size crystallites. Thus the amorphous state is seen as possessing permanent local structure, differentiating it from a glassy state.^1^ In conjunction with this, we illustrate how electronic spectroscopy of vapour deposited films may be used to characterize the electric fields which arise spontaneously in such solids, the so‐called ‘spontelectric effect’.^2^ It is further shown how a knowledge of the values of these fields leads to estimates of the size of crystallites in these materials.

Using vapour‐deposited solid ammonia, the present work involves films laid down at temperatures, T_d_, of 20 K to 80 K. A low temperature phase, T_d_=20 K to 50 K, has previously been identified as amorphous.^3^, ^4^ We show below that such films are in fact composed of crystallites of dimension >1.58 nm. At higher deposition temperatures, T_d_≥55 K, the material is designated crystalline and the corresponding crystallites are, for example, of dimension 4.73 nm at T_d_=65 K declining to 2.45 nm at T_d_=55 K. Essentially, we suggest a quantitative description of crystallinity, based on the presence of crystallites of measureable size or of known minimum size. In addition, since the two different phases are suggested to be crystalline, solid ammonia is classified as polymorphic.

We make use of the technique of synchrotron‐based solid‐state VUV spectroscopy, to identify the presence of Wannier‐Mott (WM) excitons in solid ammonia.^5^ Such excitons, familiar for many years in semi‐conductors,^6^, ^7^, ^8^ have recently been demonstrated to form in electronic excitation of high band gap, low permittivity materials, such as solid carbon monoxide (CO)^9^ and nitrous oxide (N_2_O).^10^ WM excitons consist of a bound electron‐hole pair, separated by one to a few nanometres, a dimension large compared with any intermolecular spacing. The force between the electron and hole is dominated by Coulomb attraction. The distance between the electron and hole, and thus its associated dipole moment, is sensitive to the presence of an electric field in the medium, arising from the spontelectric effect. It is this property which we exploit here.^9^, ^10^

Throughout, our understanding of our data is predicated on the basis that a WM exciton can only form if there is band structure; that is to say, there is a prerequisite for crystalline structure in the solid film for WM excitons to be found. In this connection, we note that attempts have been made to model exciton formation in inorganic amorphous solids using an effective‐mass model.^11^ Several reports have claimed to observe WM exciton formation in inorganic amorphous solids. However, it remains unclear if these excitons are supported by local domains of order, that is, small crystallites – as here – or whether WM excitons can indeed form in entirely disordered matrices.^12^ At all events, we set this on one side and note that the issue of WM exciton formation in high bandgap solids has received very much less attention than in semi‐conductors. This is due in part to the complex experimental setup required: ultra‐high vacuum, cryo‐temperatures, and the broad‐band tuneable and highly stable source of VUV photons provided by synchrotron storage ring sources.

Turning to the spontelectric effect itself, for a number of polar species, including CO^13^ and N_2_O,^14^ it is known that gas phase deposition leads to the formation of films containing spontaneous fields of typically several times 10^7^ V m^−1^ to >10^8^ V m^−1^.^2^, ^15^, ^16^ The electric field arises through spontaneous orientation of molecular dipoles, on condensation of material from the gas phase. Molecular dipoles are spontaneously oriented on deposition, yielding a surface polarisation charge of typically of 10^−4^ C m^−2^ to 10^−3^ C m^−2^, giving rise to a surface potential. This in turn gives rise to the spontelectric effect mentioned above. In the absence of any free charge, the surface potential is proportional to the thickness of the film, and therefore creates a constant spontelectric field in the film. Thus in spontelectric solids, electric fields are not imposed on the vacuum‐deposited films of material but arise naturally within them. The strength of the field depends on the temperature of deposition (T_d_) of the film, reflecting the order‐disorder nature of its origin.

The spontelectric field influences the electron and hole separation, as mentioned above, and results in a variation in absorption wavelength. Absorption spectra of films, involving WM excitons, therefore show a dependence on deposition temperature. Such variation in absorption spectra had been observed for many years, for example for CO ices,^17^ but remained unexplained until it was shown to be associated with the spontelectric effect.^9^ Thus, given a material of some crystalline character, a shift in the VUV spectra, as a function of temperature of deposition, is taken as evidence for its spontelectric nature. Here we demonstrate that cryo‐deposited ammonia films are spontelectric, observing shifts of 299 cm^−1^ between T_d_=20 K and 50 K, in the low temperature phase of solid ammonia, and 1380 cm^−1^ between T_d_=55 K and 80 K in the high temperature phase, in D, E, F and G←X Rydberg transitions between 115 nm to 130 nm. Corresponding electric fields are found here to range from 1.62×10^8^ V m^−1^ at T_d_=20 K to 2.59×10^7^ V m^−1^ at T_d_=80 K.

With regard to the quantitative definition of crystallinity, observation of a shift in absorption wavelength with deposition temperature, and thus of the temperature dependent spontelectric fields in cryo‐deposited ices, can lead to a determination of the size of nanocrystals in these ices. As the temperature of surface deposition falls, so the size of nanocrystals also in general decreases and the density and proximity of dislocations increases.^10^ In concert with this, the WM excitons expand in size at lower temperature, given that, as is generally the case, the spontelectric field is larger at lower deposition temperatures, as here.^2^ At some deposition temperature, the increased size of the excitons and the decreasing size of the nano‐crystals may come into conflict. The weakly bound electrons are then trapped at the structural defects associated with the boundaries of the crystallites.^10^, ^18^, ^19^ Thus, these ‘grain boundaries’ act as a blockade to the further expansion of WM excitons, which would otherwise take place under the greater spontelectric field at lower deposition temperature. This in turn prohibits any decrease of absorption wavelength with lower deposition temperature. Thus, while a decrease in absorption wavelength is found with decreasing deposition temperature, this may cease at some characteristic temperature. An example is the 130 nm D‐band of N_2_O, which achieves a constant minimum wavelength of absorption at 52 K and below, as shown in Figure 4 of ref [10]. Data were used to yield a characteristic crystallite diameter in solid N_2_O at 52 K of 2.5 nm, or 42±11 FCC unit cells in an average crystallite.

The phenomena associated with the effects of applied electric fields on the optical properties of high bandgap materials has been very little studied.^10^ This contrasts with semi‐conductors, for which the influence of electric fields have been very extensively investigated since the 1980s and earlier.^5^ More recently, it has been shown that applied fields give rise to electro‐optical behaviour in quantum wells and dots, termed quantum‐confined Franz‐Keldysh effects and quantum‐confined Stark effects.^20^, ^21^ The Franz‐Keldysh effect, in general, is a change in absorption or reflectance of a solid on application of an external electric field.^5^, ^10^, ^18^, ^22^, ^23^ This may be accompanied by a shift to lower photon absorption energies with higher applied field.^24^, ^25^, ^26^ Thus the effect which we observe here, of a shift to higher absorption energies in high band gap material, with no discernible change in reflectance or absorption, is quite distinct from the Franz‐Keldysh phenomenon. Turning to quantum‐confined Stark effects in semiconductors, again a red‐shift of the photoluminescence spectra takes place in the presence of an applied electric field,^20^ as in the Franz‐Keldysh effect. Exceptionally, for CsPbBr_3_, a blue‐shift is found, but only of ∼15 cm^−1^ for a field of 10^7^ V m^−1^,^20^ one to two orders of magnitude less than blue‐shifts observed here.

## 2 Results

**Figure 1:**
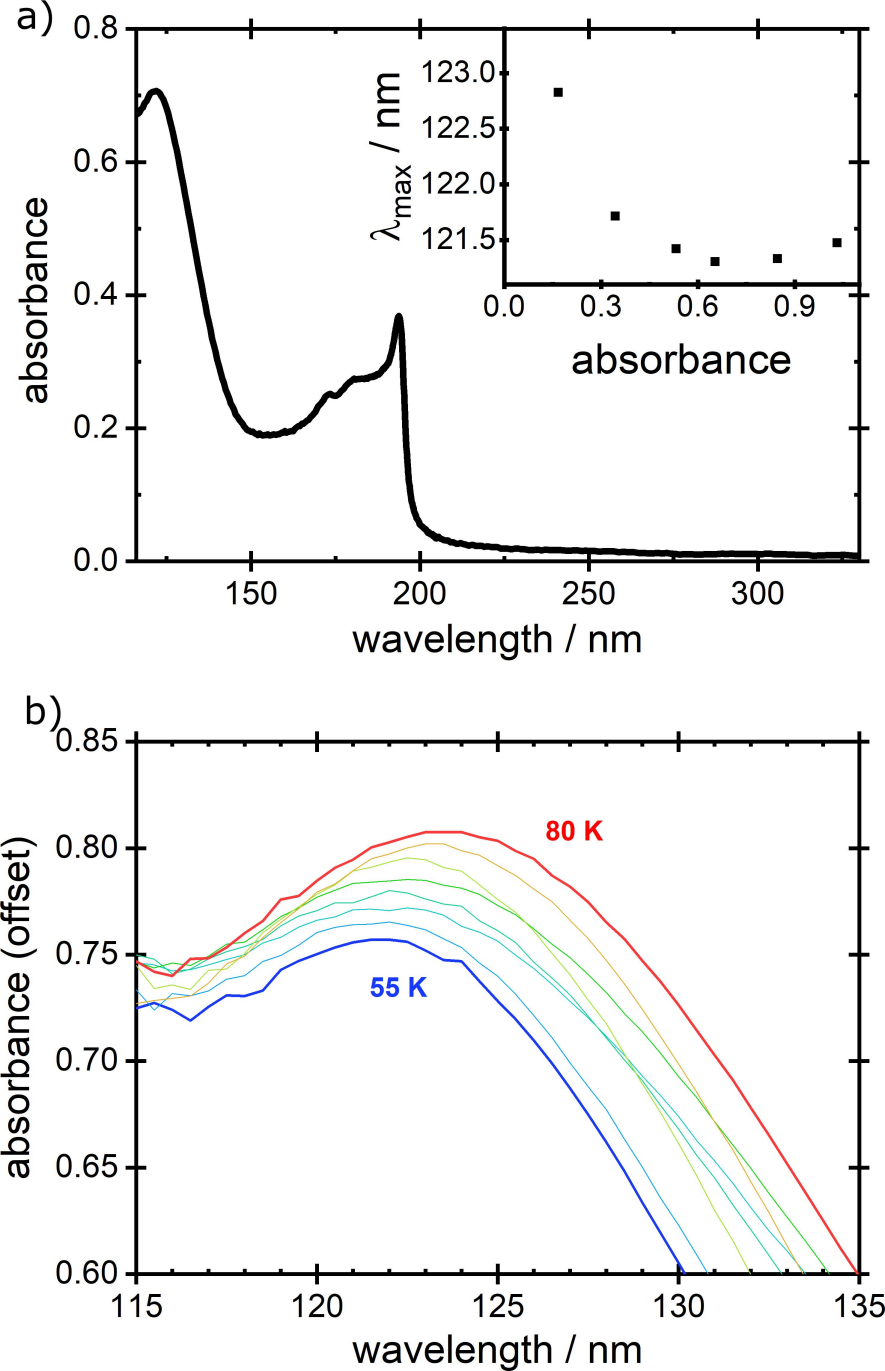
a) VUV absorption spectrum for a ∼30 ML thick NH_3_ film grown at 60 K. Inset: an example of the change in peak wavelength of absorbance between 120–125 nm as a function of increasing absorbance. For each value of T_d_, we have adopted λ_max_=the wavelength found at the lowest point of the such curves of λ vs absorbance (see text). b) The peak in absorbance, λ_max_, between 120–125 nm for films of NH_3_ grown with T_d_ between 55–80 K. Data at 55 K are aligned with the y‐axis while other data have been offset for clarity.

Films of solid ammonia were prepared as a function of thickness and temperature of deposition, T_d_, and VUV absorption spectra were recorded. Values of absorbance, *log_10_(I_0_(λ)/I(λ))*, vs *λ* were obtained and a sample spectrum, recorded at T_d_=60 K is shown in Figure 1a. We focus here upon the data for the unresolved exciton peak around 120.8 nm to 123.5 nm, which may be assigned, by comparison with gas phase spectra,^27^ to vibrational progressions in overlapping Rydberg transitions in the D, E, F and G←X transitions involving nd and ns series. Throughout, we group all these transitions together and treat the absorption as representative of a single excitonic event. This broad peak is clearly discernible at all deposition temperatures between 20 K and 80 K. A second exciton feature at 194 nm, associated with the Rydberg A←X transition emerges only at deposition temperatures ≥50 K and will be considered in a separate publication. The change in peak position with deposition temperature above 55 K is shown in Figure 1b.

**Figure 2:**
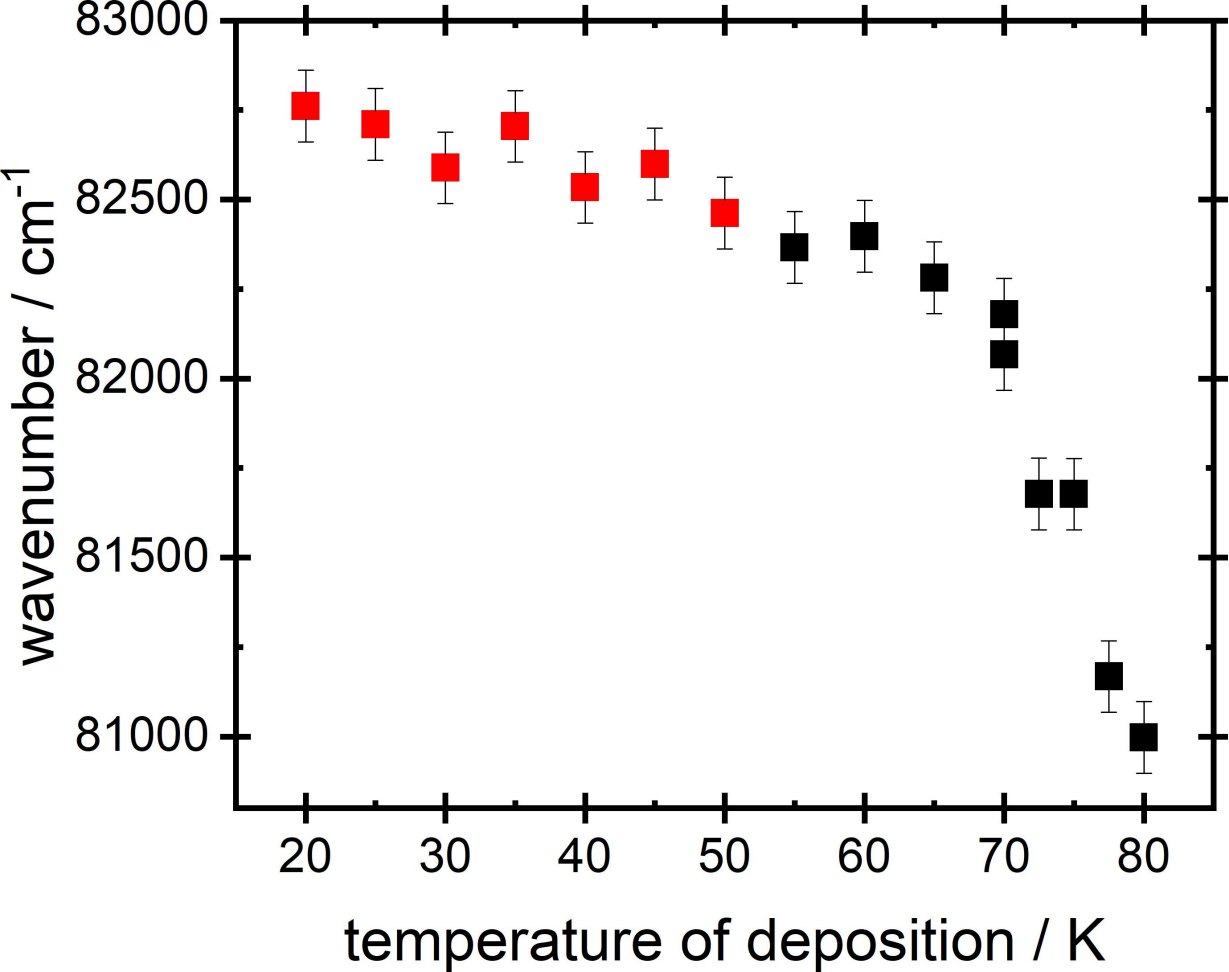
The change in the peak position, λ_max_, for films of ammonia prepared with T_d_ between 20 K–80 K. Data with T_d_=20 K–50 K represent ammonia in the low temperature phase (red squares), and data with T_d_=55 K–80 K represent ammonia in the high temperature phase (black squares).

The absorption peak that appears between 120–130 nm was fitted for the full temperature range, of 20 K to 80 K, and wavelengths (in cm^‐1^) of the maxima are shown in Figure 2 and listed in Table S1. In Table S1 and Figure 2, we discriminate between a low and a high temperature phase of solid ammonia. We have adopted the result, reported in ref [3] that there exist two distinct phases of solid NH_3_ formed through gas phase deposition. This was established in ref [3] using IR spectroscopy, showing that warming reveals the conversion of a low temperature ‘amorphous’ phase into a cubic phase at 57 K. We find a phase change between low temperature and high temperature phases at 50 K to 55 K, as observed here using a set of separate deposition experiments. Data in ref [3], based upon warming of films, locates a temperature 5 to 10 K higher, underlining the difference between warming to establish a phase change and using a set of depositions at specific temperatures. To check this, we have conducted a warming experiment, which confirms the results of ref [3] showing an abrupt shift in wavelength between 55 K and 60 K. Data are shown in supplementary materials.

The wavelengths recorded in Figure 2 refer to spectra with a maximum absorbance of ∼0.8, equivalent to a film thickness of ∼30 ML.

First, we establish qualitatively that NH_3_ ices are spontelectric. There are two characteristics of the data which demonstrate the spontelectric nature of ammonia films, first, the observation of a variation of peak wavelength, λ_max_, of absorption with deposition temperature, Figure 2, and, second, the variation of peak wavelength with film thickness, a typical example of which is shown in the inset to Figure 1a. More details are provided in supplementary materials.

The changes in absorption wavelength, with temperature of deposition, demonstrate that solid ammonia films exhibit the action of a spontelectric field upon a WM exciton. No other phenomenon can account for the large shifts in λ_max_, as discussed in detail in refs [9] and [10]. Briefly, other effects may involve (i) the permanent dipole moment change in ammonia associated with the Stark shift between the X and D, E, F, G states, (ii) the induced dipole moment change through polarizability changes between the X and D, E, F, G states, or (iii) changes in the average degree of dipole orientation associated with changes in the deposition temperature, T_d_, leading to differences in dipole‐dipole interaction energies at different T_d_. The discussion in refs [9,10] shows however that these phenomena can contribute only a fraction of a wavenumber to the spectral shift.

## 3 Interpretation of Results

We now derive the electric fields that may be found in ammonia films as a function of deposition temperature. This requires two models, the first of which is for the effect of an electric field upon WM excitons.^9^, ^10^ This is outlined in Section 3.A. The second model, for the spontelectric effect itself and described in detail in ref [2] is outlined in Section 3.B.

Having established the electric fields in the solid films, we are then able to show that the low temperature phase, formed between 20 K to 50 K, contains excitons, which are of dimension half to one third of the size of those found in the high temperature phase, formed at ≥55 K. As mentioned in the introduction, this also enables us to identify the size of crystallites in the high temperature phase at 65 K, 60 K and 55 K, approaching the phase change, which lies between 50 K and 55 K. An estimate can also be made of the minimum size of crystallites in the low temperature phase.

### 3.A The Exciton Model

A model for the effect of an electric field upon the absorption wavelength of a WM exciton has been described in detail in refs [9, 10]. Our analysis shows how λ_max_ varies with electric field. The assumptions of this model are: (i) that the electron‐hole separation is much greater than the separation between molecules in the crystal and, following from this; that (ii) the electrical permittivity, ϵ, may be assumed to be that of the bulk material, given by n^2^ for values of n between 20 K and 100 K,^28^ where n is the refractive index of solid NH_3_. This yields ϵ=2.19±0.145. In addition, we assume that the only significant mutual interaction between the hole and the electron is the Coulomb attraction.

Writing the electron‐hole separation, in the hypothetical absence of the spontelectric field, as r and using atomic units throughout (e. g. ϵ_0_=1/(4π), electronic charge=1), the force between the hole and electron =1/r^2^. In the presence of the spontelectric field, E_Sp_, r increases to r+Δr and the force becomes 1/(r+Δr)^2^. Since E_Sp_ acts on both the hole and electron, there is an additional force of 2 E_Sp_. This is balanced by the change in coulombic force and thus 1/(ϵ r^2^)−1/[ϵ (r+Δr)^2^]=2 E_Sp_, yielding:

(1) $$\Delta r=r[{\left(1-2E_{Sp}\epsilon \;r^{2}\right)}^{-\frac{1}{2}}\;-1]$$

The hole‐electron pair constitute a dipole of magnitude 2(r+Δr). Given that the torque on the WM dipole causes it to align with the spontelectric field, the change in energy of the system is given by the change in the dipole, 2Δr, multiplied by the ambient electric field =2ΔrE_Sp_. This energy change, associated with any deposition temperature and thus spontelectric field, corresponds to λ_0_^−1^–λ_max_^−1^, where λ_0_ is the wavelength of absorption in the absence of a spontelectric field. Given values of λ_0_ and r, we are then able to evaluate how E_Sp_ changes with deposition temperature.

An immediate problem, however, is that λ_0_ and r are unknown quantities at this stage, although initial estimates of values of λ_0_ may be made from experimental data (see supporting materials and Section 3.B). We therefore confront an ill‐posed problem, whose solution is to appeal to additional knowledge, so‐called regularization. Regularization is achieved using a well‐established mean‐field model of the spontelectric effect itself, described in Section 3.B.

### 3.B The Spontelectric Model and Regularization

The semi‐empirical model outlined here has been shown to reproduce the temperature dependence of observed spontelectric fields in solid films of nitrous oxide, nitrous oxide diluted in xenon, cis‐methyl formate (cis‐MF) and CCl_x_F_y_ (x+y=4).^2^ The model also underpins a description of the observed deposition temperature dependence of IR spectra of films of N_2_O, cis‐methyl formate, and CO.^29^, ^30^, ^31^, ^32^

Recollecting that the spontelectric field arises through dipole orientation, we define this degree of orientation as <μ>/μ_0_, where μ_0_ is the dipole moment of the ammonia in the solid state and <μ> is its average component in the z‐direction, normal to the plane of the film. The mean‐field model expresses a local electric field in the z‐direction, E_z_, at any moiety in the solid, in terms of three unknown parameters, <E_S_>, <E_A_> and ζ as follows:

(2) $$E_{z}=<E_{S}>\left[1+\zeta {\left(<\mu >/\mu_{0}\right)}^{2}\right]-<E_{A}>\left(<\mu >/\mu_{0}\right)\;$$

where <E_S_>, <E_A_> and ζ are taken to be independent of deposition temperature, over any temperature range for which there is no structural modification associated with a phase change. Thus, here we seek two sets of such parameters for T_d_=20 K to 50 K and T_d_=55 K to 80 K, for the two phases of solid NH_3_. The magnitude of the net spontelectric field in the material is given by E_Sp_=<E_A_><μ>/μ_0_.

Mean field theory gives an implicit expression for <μ >/μ_0_, yielding the familiar Langevin function for orientational order opposed by thermal disorder:^5^

(3) $$\frac{\left\langle \mu \right\rangle }{\mu_{0}}=\coth\left(\frac{E_{z}\mu_{0}}{T_{d}}\right)-{\left(\frac{E_{z}\mu_{0}}{T_{d}}\right)}^{-1}$$

The solid state dipole moment μ_0_ is expressed as μ_g_/(1+αk/s^3^),^2^, ^33^ where s is the average spacing between successive layers, k=11.034,^34^ μ_g_ is the gas phase dipole moment of the molecule involved and α is the molecular polarizability. Values for NH_3_ are shown in Table 1.

**Table 1:** Parameters for NH_3_.

Polarizability, α^[a]^	2.158×10^−30^ m^3^
Layer spacing, s^[b]^	0.25365 nm
Gas phase dipole moment, μ_g_	1.42 D
Dipole moment in the solid state, μ_0_	0.577 D

[a] α is the isotropic molecular polarizability. [b] The value of the layer spacing, s, is taken from X‐ray and neutron scattering data.^35^, ^36^

The spontelectric state may be viewed as a classic order‐disorder system, in which dipole orientation is countered by thermal fluctuations. Thus, Equation 2 may be inserted into Equation 3 to yield:

(4) $$<\mu >/\mu_{0}=\;-T_{d}/\left\{\mu \left[<E_{S}>\left(1+\zeta {\left(<\mu >/\mu_{0}\right)}^{2}\right)-<E_{A}>\left(<\mu >/\mu_{0}\right)\;\right]\right\}+coth\left\{\mu \left[<E_{S}>\left(1+\zeta {\left(<\mu >/\mu_{0}\right)}^{2}\right)-<E_{A}>\left(<\mu >/\mu_{0}\right)\;\right]/T_{d}\right\}$$

For the low temperature regime, Equation 4 may be rewritten, using coth(x)–x^−1^∼x/3, to yield an explicit Equation for <μ >/μ_0_ as a function of deposition temperature:

(5) $$<\mu >/\mu_{0}=\;\left\{\chi -\;\left[\chi^{2}\;-\;4{<E_{S}>}^{2}\mu^{2}\zeta \;\right]{}^{1/2}\right\}/\left(2<E_{S}>\mu \zeta \right)$$

with an accuracy of ∼0.1 %, where χ=$<E_{A}>\mu \;+3T_{d}$ . For the high temperature regime, with $T_{d}$ ≥55 K, coth(x)–x^−1^∼x/3 is no longer satisfied and the implicit Equation (4) must be solved numerically to yield a set of $<\mu >/\mu_{0}$ vs $T_{d}$ .

The criterion which we seek to satisfy is that spontelectric fields, E_Sp_, as a function of deposition temperature, and the corresponding values of <μ>/μ_0_, are consistent with both the exciton model and the spontelectric model, obeying E_Sp_=<E_A_><μ>/μ_0_. That is to say, we seek the set of parameters, <E_S_>, <E_A_>, ζ, λ_0_ and r, which simultaneously satisfy values of E_Sp_ derived from the observations of spectral shift, where the shift is given by 2Δr.E_Sp_ (Section 3.A), and the values of <μ>/μ_0_ and E_Sp_ given by the model embodied in Equations 2–5. We make the assumption, borne out by the numerical experiments, that λ_0_ and r are sufficiently weakly correlated that they can be independently determined. In addition, we have experimentally based estimates of λ_0_.

On the basis of some chosen values of λ_0_ and r, we find a value of a set of spontelectric fields vs deposition temperature. Any such set, for an assumed value of <E_A_>, dictates a set of values of <μ>/μ_0_. For this value of <E_A_> and some choice of <E_S_> and ζ, Equation 4 or 5 then returns a new set of values of <μ>/μ_0_. We require that these two sets of values of <μ>/μ_0_ agree and, when they do so, we have identified an acceptable set of values of <E_S_>, <E_A_>, ζ, λ_0_ and r. Thus we choose various values of λ_0_ and r and establish values of <E_S_>, <E_A_>, ζ, λ_0_ and r which satisfy this self‐consistency, on a least‐squares basis. This procedure regularizes the ill‐posed problem. Knowing the values of <E_S_>, <E_A_> and ζ, we are then able to calculate the spontelectric fields as a function of deposition temperature, as shown below.

Starting values of λ_0_ in this iterative process were obtained from experimental data such as those in the inset of Figure 1a, for very thin films of absorbance of ∼0.1; see discussion in supporting materials. Averaging over a large number of data, yields starting values of λ_0_=123.9±1 nm for the low temperature phase and λ_0_=124.2±0.66 nm for the high temperature phase (2 SD). Iteration, in conjunction with values of r as described above, yielded figures of respectively 122.85±0.15 nm and 124.0±0.15 nm. These values of λ_0_ enable estimates to be made of the shifts, from one value of T_d_ to another, of exciton absorption wavelength, λ_max_. These shifts are shown in Table S1, columns 3 and 6. Turning to the value of r, the electron‐hole separation in the putative absence of a spontelectric field, the only constraint put upon the value of this quantity is that it should be considerably greater than the intermolecular spacing of 0.254 nm.^35^ Derived values are r=1.06±0.11 nm and 2.38±0.13 nm for the low and high temperature phases respectively. Derived values of <E_S_>, <E_A_>, ζ are given in Table 2.

**Table 2:** Derived spontelectric parameters for NH_3_.

Parameter	Low‐temperature phase T_d_=20 K to 50 K	High‐temperature phase T_d_=55 K to 80 K
r	1.06±0.11 nm	2.38±0.13 nm
λ_0_	122.85±0.15 nm	124.0±0.15 nm
<E_S_>	1.762×10^8^ V m^−1^	1.609×10^7^ V m^−1^
<E_A_>	5.272×10^9^ V m^−1^	5.312×10^9^ V m^−1^
ζ	9.728	2.117×10^4^

With reference to Table 2, note that the theoretical model of spontelectrics requires that <E_A_>=μ_0_/(ϵ_0_Ω).^2^ An experimentally based estimate of Ω, the molecular volume, may be obtained via the known polarizability of ammonia, to within ∼10 %.^37^ This gives <E_A_>=5.304±0.48×10^9^ V m^−1^ which may be compared with values respectively of 5.272×10^9^ V m^−1^ and 5.312×10^9^ V m^−1^ in Table 2, for the low and high temperature phases, obtained by fitting. This serves as a useful independent check on values of <E_A_> derived from the spontelectric model and the regularization procedure, obtained without any restriction that <E_A_>=μ_0_/(ϵ_0_Ω).

**Figure 3:**
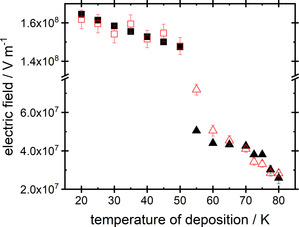
Spontelectric fields, E_Sp,_ in solid films of NH_3_ as a function of deposition temperature. Black points are values derived from the observed spectral shifts, and arrive as outputs from the exciton‐model. Red points are values derived through application of the theoretical model for the spontelectric state, using the parameters in Table 2 relevant to this model. Squares represent T_d_ associated with the low temperature phase and triangles represent T_d_ associated with the high temperature phase. Note that the y‐axis is split between the two phases. Where error bars are not visible they are smaller than the data point. Note the discrepancy between observed and model values at 60 K and 55 K.

The consistency and effectiveness of the regularization achieved may be assessed by comparing the two sets of spontelectric field vs deposition temperature, derived from (i) spectroscopic data and (ii) from the model embodied in Equations 2–5, using the values of parameters shown in Table 2. Results are shown in Figure 3.

**Table 3:** Characterization of the crystallite size in solid ammonia approaching the phase change at 50–55 K.

Temperature of deposition, T_d_ /K	Size of crystallite, nm ±8%.	Number of unit cells in a crystallite	Number of molecules of NH_3_ in the crystallite
65	4.727	422 (+98, −85)	1688 (+392, −340)
60	3.620	189 (+44, −38)	756 (+176, −152)
55	2.450	59 (+16, −13)	236 (+64, −52)

Significant errors in values of the degree of orientation, obtained using Equations 4 and 5, arise indirectly through uncertainties in the values of r and λ_0_ (Table 2). We estimate that the uncertainties in shifts shown in Table S1 are ±100 cm^−1^, where this arises from the fitting procedure for λ_max_; supplementary materials. These errors translate into ∼10 % uncertainties in <E_S_>, <E_A_>, ζ, which then promulgate into uncertainties in derived spontelectric fields associated with the red points in Figure 3. Values of field derived from spectral shifts, the black points in Figure 3, are generally much less sensitive to uncertainties in measured shifts, but sensitivity increases at high deposition temperature for which shifts are small, at T_d_=77.5 K and 80 K. Results in Figure 3 are discussed in Section 4, with particular reference, in Section 4.B, to the discrepancy between values of spontelectric fields derived from the theoretical model of the spontelectric state and those derived from spectral shifts in the range T_d_=55 K to 60 K.

**Figure 4:**
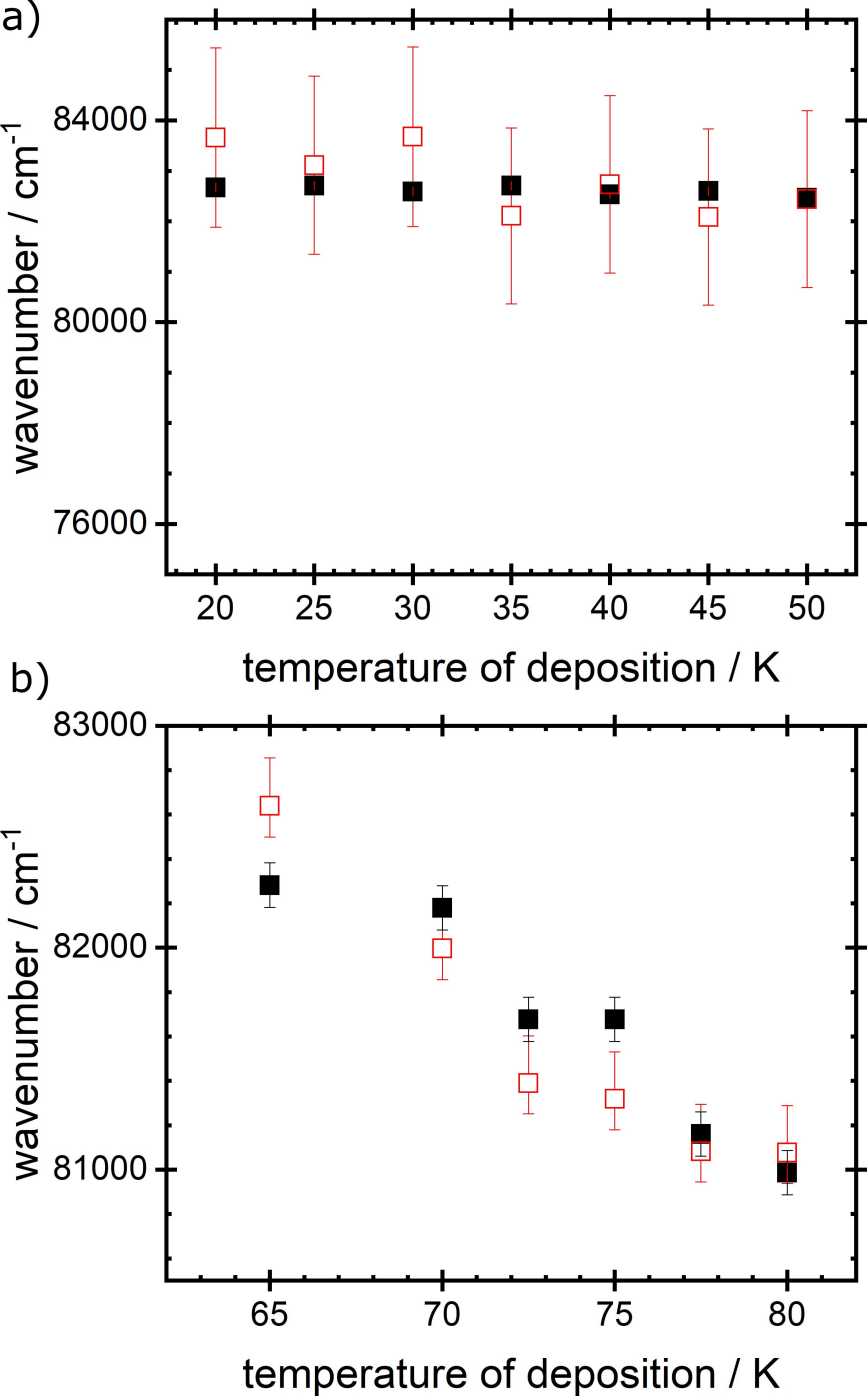
Experimental (▪) and calculated (□) values of the peak wavelength of absorption of solid NH_3_ as a function of deposition temperature, T_d_. Calculated values were obtained using the parameters shown in Table 2.

A direct comparison may be made between the observed variation with deposition temperature of values of λ_max_ (Figure 2) and those predicted on the basis of values of parameters shown in Table 2. This is shown in Figures 4a and b, for the low and high temperature phases respectively. At T_d_=55 K and 60 K, values of Δr are no longer given by Equation 1, because a blockade in the expansion of the exciton results from the restricted size of crystallites, Section 4.B. Thus in Figure 4b, comparison with experiment is shown only between T_d_=65 K and 80 K.

## 4 The Nature of the Two Phases of Solid NH_3_ and the Physical Dimensions of Crystallites

### 4.A The spontelectric effect in the low temperature and high temperature phases

For the low temperature phase, the spontelectric field falls by only ∼10 % between T_d_=20 K and 50 K. The variation of the spontelectric field with T_d_ is principally determined by the interplay between the value of the term <E_S_>ζ (<μ>/μ_0_ )^2^ in Equation 2, relative to <E_S_>, and the spontelectric field itself, <E_A_><μ>/μ_0_. This arises since <μ>/μ_0_ is the single temperature dependent quantity in the model, for any phase, and determines the temperature dependent cross‐talk between the spontelectric field and the field impinging on the molecular constituents. In the low temperature phase, the absolute value of <E_S_>ζ (<μ>/μ_0_)^2^ is ∼1 % of <E_S_>. This low figure makes for a weak temperature dependence of the spontelectric field.

By contrast, for the high temperature phase, the spontelectric field drops by ∼50 % between T_d_=55 K and 80 K. Values of <E_S_>ζ (<μ>/μ_0_)^2^ lie between equal and twice the value of <E_S_>, as opposed to ∼1 % of <E_S_> for the low temperature phase. As we show in Section 4.B, the essential difference between the low and high temperature phases is that crystallites at (say) 20 K contain considerably fewer ammonia molecules than a crystallite at (say) 65 K. It is possible the observed behaviour stems from the much higher proportion of molecules associated with grain boundaries, and associated lack of order, in the low temperature phase compared with the high temperature phase. Quantum chemistry simulations may shed light on this suggestion.

### 4.B Physical dimensions of the exciton in solid NH_3_ as a function of deposition temperature.

We estimate the size of nanocrystals in ammonia by recognizing that the electrons associated with the exciton become trapped at grain boundaries, instituting a blockade in the expansion of WM excitons. The size of any exciton is given by r+Δr, where Δr is given by Equation 1.

For the low T_d_ range of 20 K to 50 K, there is no evidence of a blockade in the increase of the hole‐electron separation and we estimate that the size of the crystallites is >1.579 nm, the value at 20 K. However in the high temperature phase, between 55 K and 80 K, Figure 3 shows clear evidence of a blockade for T_d_<65 K. At 65 K, the size of the exciton is 4.727 nm. As the deposition temperature is reduced, so the size of crystallites shrinks and the spontelectric field increases. Data in Figure 3 are interpreted as showing that at 65 K the size of the crystallites and the size of the exciton coincide, since this is the lowest temperature at which values of spontelectric field derived from the shifts and from the spontelectric model agree. At lower T_d_, the exciton is no longer able to expand, with the electron trapped at structural defects marking the grain boundaries between crystallites. Thus, Equation 1 no longer holds. In the absence of this blockade, the spectral shifts would otherwise increase much more markedly for T_d_<65 K than is observed. Confinement of excitons due to crystallites therefore yields an explanation for the abrupt interruption of the decrease in absorption wavelength at T_d_<65 K (Figure 2). The size of the confined exciton, given by r+Δr, and therefore the crystallite size for T_d_=65 K, 60 K and 55 K are shown in Table 3 with detailed analysis given in the supplementary material.

In thin films of semi‐conductors there is a so‐called ‘dead layer’ at the surface‐vacuum interface, devoid of excitons and of thickness approximately the radius of the exciton.^38^ We see no evidence of such a layer in the present case. For example, for the high temperature phase, the radius of the exciton, r/2, is 1.19±0.06 nm (Table 2) in the absence of a spontelectric field, for a 4 ML film. The thickness of a 4 ML film is 1.02 nm and the exciton peak is nevertheless observed. We conclude that the surface does not repel excitons, in contrast to semi‐conductors. We note also that exciton dimensions shown in Tables S2 and S3 are such that they may all be accommodated in the typical 30 ML film of thickness 7.62 nm.

## 5 Conclusions

We set out to establish a clear definition of the loosely used description of solids as ‘amorphous’. The results presented here show how the term may be placed upon a quantitative foundation, defining a lower limit on crystallite size in the low temperature phase of solid NH_3_. In addition we are also able to estimate the decreasing size of nano‐crystals that form in the high temperature phase (T_d_≥55 K), as the system approaches a phase change between 50 K and 55 K.

Our results rest upon the notion that crystallites are at least as large as the size of the excitons, r+Δr above. It turns out that in the low temperature phase of solid NH_3_, formed between T_d_=20 K and 50 K, we may only state that the crystallites are greater in dimension than that of the exciton at 20 K of 1.58 nm. At higher values of T_d_, in the high temperature phase at T_d_=65 K, 60 K and 55 K, the crystallite however constrains the physical limits of the exciton. The physical foundation for this method has been established through earlier work involving VUV spectroscopy of CO and N_2_O ices.^9^, ^10^

Our conclusions may be summarized as follows:

- Spectroscopic data, combined with established models of the spontelectric state and the Stark shift of VUV spectra in spontelectric films, have been shown to be sufficient to establish the strength of electric fields within solid NH_3_.
- On the basis of estimated spontelectric fields for the low temperature phase of solid NH_3_, formed at temperatures of deposition between 20 K and 50 K, exciton dimensions are found to be smaller than the size of nano‐crystallites. This gives a lower limit on the size of crystallites in this ‘amorphous’ phase. Thus at 20 K, we identify nanocrystals composed of >(π/48).(1.58/s)^3^∼16 unit cells, that is, composed of >64 moieties.
- Spontelectric fields in the high temperature phase, between T_d_=55 K and 80 K, show that crystallites are of size 4.73 nm at T_d_=65 K, falling to 3.62 nm at 60 K and 2.45 nm at 55 K. They are correspondingly comprised, to ±20 %, of 1688, 756 and 236 moieties. In this temperature regime, the perimeters, or grain boundaries, of nanocrystals constitute a blockade to the expansion of excitons under the influence of the spontelectric field.

## Conflict of interest

The authors declare no conflict of interest.

## Supporting information

As a service to our authors and readers, this journal provides supporting information supplied by the authors. Such materials are peer reviewed and may be re‐organized for online delivery, but are not copy‐edited or typeset. Technical support issues arising from supporting information (other than missing files) should be addressed to the authors.

Supplementary
