# Supplementary material for: Crystallites and Electric Fields in Solid Ammonia
Source: ChemistryOpen. 2020 Jul 16;9(10):983–90. doi: 10.1002/open.202000118 (PMC7528761; doi:10.1002/open.202000118)
Supplement: Supplementary file 1 — Supplementary [file OPEN-9-983-s001.pdf]

ChemistryOpen

Supporting Information

Crystallites and Electric Fields in Solid Ammonia

Andrew Cassidy,* Rachel L. James, Anita Dawes, and David Field

A. Experimental Section

Ammonia ices were deposited on a MgF_2 substrate in an experimental chamber with a base pressure of 10^{-9} mbar, attached to the AU-UV beam line on the ASTRID2 synchrotron storage ring at the Institute for Physics and Astronomy, Aarhus University, where all data were taken. The MgF_2 substrate was held in thermal contact with a cold finger, cooled by a closed cycle helium cryostat, yielding a base temperature of 20 K. The substrate temperature could be set at will and controlled to within ±0.1 K, using an Oxford Instruments controller (ITC502). Ammonia gas (99% purity) was introduced through a 3 mm nozzle, placed 2 cm from the substrate. Films were grown at substrate temperatures between 20 K and 80 K, the latter the highest value at which stable films could be formed, given the base pressure. Film growth was monitored using a He-Ne laser interference technique[1,2] enabling control of film thickness, d, via $d = t\lambda_l/(4w.n\cos\theta)$ where t is the total deposition time, λ_l is the wavelength of the laser = 632.8 nm, w is the half-period of the oscillation of the optical signal, n is the refractive index of solid NH_3 = 1.48[3] and θ is the angle of incidence of the laser beam upon the sample. Films were laid down at a rate of typically 0.37 nm s^{-1} or 1.46 monolayers (ML) s^{-1}, given a layer spacing of 0.254 nm.[4]

The AU-UV beamline provides tuneable plane-polarized VUV, with a high-energy cut-off at 10.78 eV (115 nm) via a LiF entrance window and MgF_2 exit window. The beamline was operated with a resolution of 0.075 nm throughout. Background spectra, $I_0(\lambda)$, were recorded as a function of incident wavelength λ. Values of the absorbance, $log_{10}(I_0(\lambda)/I(\lambda))$, vs λ were obtained, where $I(\lambda)$ is the transmitted intensity through the film and substrate. A typical spectrum of a film deposited at 60 K, for the entire range of wavelengths between 115 and 320 nm, is shown in Figure 1. For any deposition temperature, between five and ten spectra were recorded, each with an increasing thickness such that the absorbance varied between 0.1 and ≥1. This was repeated for each of 15 different deposition temperatures between 20 and 80 K.

B. Determination of absorption wavelengths

In order to find the centre of the peak responsible for the absorption between 120-125 nm in Figure 1 in the main text, data were fitted with two gaussians, one centred at the absorption peak, and a second at lower wavelengths to take account of a rise in absorption, just below the cut-off.

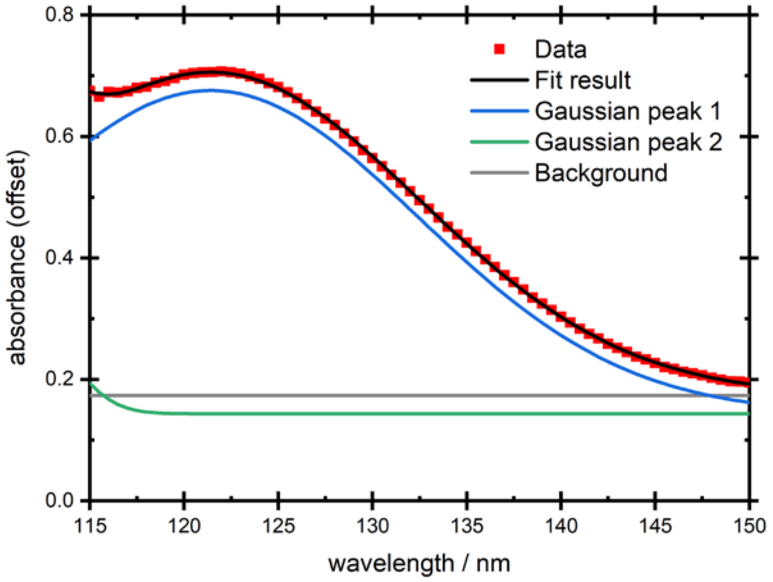

Figure A1: An example of a fit to the absorption at 125 nm, using 2 gaussians, for T_d = 60 K

Table S1. Fitted wavelengths, λ_{max}, vs temperature of deposition for the two phases of solid NH_3.

Low temperature phase				High temperature phase		
Deposition temperature, T_d	Wavelength/nm λ_{max} ±0.145 nm	Shift /cm^{-1}		Deposition temperature, T_d	Wavelength/nm λ_{max} ±0.145 nm	Shift /cm^{-1}
20	120.8294	1361.22		55	121.4085	1721.44
25	120.9043	1309.92		60	121.3633	1752.04
30	121.0817	1188.82		65	121.5332	1636.84
35	120.9120	1304.72		70	121.6847	1534.44
40	121.1621	1134.02		72.5	122.4328	1032.34
45	121.0661	1199.42		75	122.4331	1032.14
50	121.2679	1062.02		77.5	123.2125	515.44
				80	123.4774	341.34

The shift, col.3 and 6, is given by λ^{-1} - λ_0^{-1} in cm^{-1} where λ_0 is the wavelength associated with zero spontelectric field: see Section A in the main text. The values of absorbance at which wavelengths were recorded are discussed in the main text in relation to Fig. 1 and in section D below. Low and high temperature phases are discussed in Section C below. An error of ±0.145 nm corresponds to ±100 cm^{-1}.

C. Phases of solid ammonia

As described in [5] and confirmed here, there are two distinct phases of solid NH_3. These are associated here with a deposition temperatures of T_d = 20 K to 50 K and T_d = 55 K to 80 K. Our current experiments yield a phase change between 50 K and 55 K, a few degrees lower than the Figure of 57 K reported in [5]. Data in [5] were obtained through warming of the material, as opposed to a set of independent experiments carried out at different deposition temperatures, as here. We have performed a warming experiment, equivalent to that in [5] and this indeed yields a temperature for a phase change of between 55 K and 60 K, in agreement with [5]. These data are shown in Figure A2, with a

clear change in lineshape evident between experiments at 55 K and 60 K. Films were deposited at 20 K and then successively warmed to the temperatures shown.

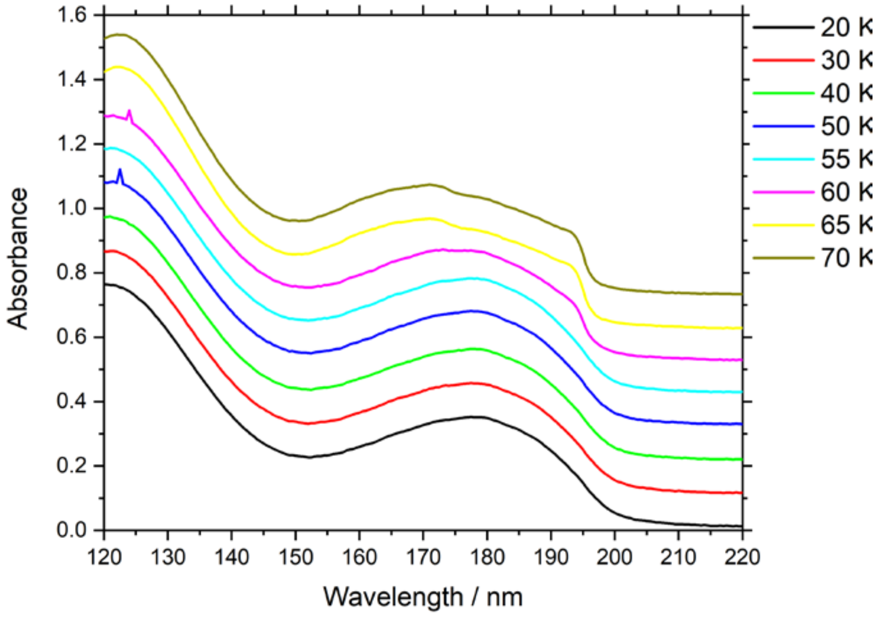

Figure A2. VUV absorption spectra of a ~30 ML film of solid NH_3 deposited on a MgF_2 window, obtained at the VUV beamline on the ASTRID2 storage ring. The clear change of the form of the spectrum in 195 nm region, and other less prominent changes, on warming between 55 K and 60 K is taken as evidence of a phase change in this temperature range, as reported in [5]. Data at 20 K show a maximum absorbance of 0.8 and all other data are offset for clarity.

D. Choosing the appropriate film thickness to assign λ_{max}

We consider here the data in the inset to Figure 1 in the main text. These, and similar data for all values of T_d, furnish qualitative proof of the presence of the spontelectric effect in solid ammonia. The characteristic of the spontelectric state is that it is a many-body, non-local phenomenon. For the spontelectric state to form, these properties require the presence of a film of sufficient thickness. Observations show in general that as the film thickness is increased beyond a few ML, the spontelectric field progressively assumes its full strength.[6] Data, such as those in the inset, show that the peak absorption wavelength moves to a shorter value as film thickness is increased. The developing field acts accordingly to modify the effective electron-hole separation and therefore to augment the dipole associated with the exciton. This leads to an augmented Stark blue-shift. As the film thickness is further increased, the wavelength is observed to level out to some minimum value, corresponding to a fully developed spontelectric field. Thus the data in the inset, typical of all temperatures of deposition investigated, reflect the known behaviour of the spontelectric field with film thickness.

Data of the form shown in the inset also provide values of the wavelength of absorption, λ_0, associated with a putative zero spontelectric field. Such values are required for quantitative analysis of our data (section A in the main text). Referring once more to the

inset in Figure 1, an absorbance of ~0.1 corresponds to film of thickness of ~4 ML, following the calibration described in section A, above. For such films, the exciton peak around 120 nm however remains prominent. These films are therefore in a regime in which there is a defined lattice, with associated band structure to form WM excitons. 4 ML films are not however of sufficient thickness to be spontelectric. Therefore the corresponding value of absorption wavelength gives an estimate of the value of λ_0, the absorption wavelength in the absence of the spontelectric field (section A2 in the main text). Data in the inset also indicate the correct choice of absorbance, of ~0.6 to ~0.8, used to record the wavelengths, λ_{max} (Figures 2, in the main text and Table S1, above). Note that onset of saturation, for absorbance >0.8, causes a distortion of the peak and can lead to incorrect estimates of λ_{max}.

E. Physical dimensions of the exciton in solid NH_3 as a function of deposition temperature

Data at 65 K, 60 K and 55 K may be used to estimate the size of the crystallites at these temperatures. The sizes of the excitons, $r + \Delta r_{65}$, $r + \Delta r_{60}$ and $r + \Delta r_{55}$, are then postulated to be the sizes of crystallite at these deposition temperatures. Essentially we now ask what sizes, $r + \Delta r$, for any pair of temperatures, is necessary to give the observed shift for that pair of temperatures, as in [7] in which a similar blockade is observed. Given that the change in energy of the system in the presence of the spontelectric field = $2\Delta r.E_{Sp}$ (section A.1 – main text), the shift in energy, $\Delta\mathcal{E}_{ij}$, between any two temperatures, T_1 and T_2 is given by

$$\Delta\mathcal{E}_{ij} = 2[(r + \Delta r_{T1})\, E_{T1} - (r + \Delta r_{T2})\, E_{T2}] \qquad \text{Eq. S1}$$

Values of $\Delta\mathcal{E}_{ij}$ are given in Table S1 in section B. Using values of T_2 = 70 K to 80 K, and T_1 = 55 K or 60 K, and averaging over the results for Δr, yields $\Delta r_{60} = 1.24\pm0.22$ nm and $\Delta r_{55} = 0.07\pm0.17$ nm. The latter value indicates that expansion of the exciton is essentially completely inhibited under the influence of the spontelectric field at 55 K. Thus the crystallite size decreases from 4.73±0.34 nm at 65 K to 3.62±0.26 nm at 60 K to 2.45±0.21 nm at 55 K, Table S2, where as noted, the crystallite size at 55 K is, within the errors, equal to the value of r = 2.38±0.13 nm, the dimension of the exciton unperturbed by the spontelectric field, Table 2, main text.

We now ask, how many unit cells are there contained in crystallites of solid ammonia and how does this number change as we approach the phase change at 50 to 55 K? The structure of solid ammonia is close to face-centred cubic.[4,8] Below, we use the working hypothesis that the structure of solid ammonia remains FCC throughout our T_d = 20 K to 80 K temperature range, noting that data taken at 2 K and 77 K support this.[8] We recognize however that there is some structural change to the unit cell which leads to the disappearance of the exciton at 195 nm at T_d < 55 K and to the formation of smaller crystallites in the low temperature range. Hence the polymorphism in solid ammonia. The layer spacing, s, in an FCC lattice is half the dimension of the unit cell, giving a unit cell volume = $8s^3$. The exciton volume, assumed spherical and of diameter $r + \Delta r$, has a value of $\pi (r + \Delta r)^3/6$. The number of unit cells within this volume is therefore given by $(\pi/48)\,[(r + \Delta r)/s]^3$. If s = 0.254 nm, the number of unit cells lies between 46 and 75, with a mean of 59

at 55 K. There are 4 molecules per unit cell in a FCC lattice. This analysis therefore gives estimates for the number of molecules vs deposition temperature involved in a regular defect-free conformation, that is, in a crystallite, in NH_3 films. Thus at 65 K, the number of molecules is 1688, at 60 K; 756 and at 55 K; 236. These results are collated in Table 3 in the main text. At 20 K, we have seen that the size of the crystallites in the low temperature phase is >1.579 nm, that is, >~16 unit cells or containing >64 molecules.

Table S2: Crystallite size in the low temperature phase of solid NH3

Deposition temperature, T_d	Spontelectric field[a] /10^8 V m^{-1}	Size of exciton[b] /nm
20	1.618	1.579
25	1.596	1.567
30	1.542	1.536
35	1.594	1.566
40	1.516	1.522
45	1.547	1.539
50	1.479	1.503

[a]Electric field uncertainties are shown in Fig. 3, main text. [b]The diameter of the exciton, r + Δr, equal to the lower limit of the size of crystallites in this phase. Absolute errors: $\pm$7%.

Table S3: Crystallite size in the high temperature phase of solid NH_3.

Deposition temperature, T_d	Spontelectric field[a] /10^7 V m^{-1}	Size of exciton[b] /nm
55	7.188	2.450
60	5.054	3.620
65	4.326	4.727
70	4.255	4.617
72.5	3.806	4.063
75	3.806	4.063
77.5	3.019	3.439
80	2.586	3.199

[a]Electric field uncertainties are shown in Fig. 3, main text. [b]The size of the exciton, r + Δr. Note that for 60 K and 55 K, the size of the exciton is estimated using equation S1, as detailed in the text. Absolute errors: $\pm$8%.

References

[1] A. Dawes, R. J. Mukerji, M. P. Davis, P. D. Holtom, S. M. Webb, B. Sivaraman, S. V. Hoffmann, D. A. Shaw, N. J. Mason, *J. Chem. Phys.* **2007**, *126*, 244711.
[2] M. S. Westley, G. A. Baratta, R. A. Baragiola, *J. Chem. Phys.* **1998**, *108*, 3321–3326.
[3] M. Á. Satorre, J. Leliwa-Kopystynski, C. Santonja, R. Luna, *Icarus* **2013**, *225*, 703–708.
[4] I. Olovsson, D. H. Templeton, *Acta Cryst* **1959**, *12*, 827–832.
[5] W. Zheng, R. I. Kaiser, *Chemical Physics Letters* **2007**, *440*, 229–234.

[6] D. Field, O. Plekan, A. Cassidy, R. Balog, N. C. Jones, J. Dunger, *International Reviews in Physical Chemistry* **2013**, *32*, 345–392.
[7] A. Cassidy, R. L. James, A. Dawes, J. Lasne, D. Field, *Phys. Chem. Chem. Phys.* **2019**, *21*, 1190–1197.
[8] A. W. Hewat, C. Riekel, *Acta Cryst A* **1979**, *35*, 569–571.
